# Emergency Preparedness and Response: Strategies for Mass Casualty Incidents

**DOI:** 10.7759/cureus.89158

**Published:** 2025-07-31

**Authors:** Safeer Ahmad Javid, Maheen Jamil, Bushra Noor, Muhammad Rizwan Umer, Areeba Asghar, Muddasir Reyaz Hassan, Amna Akbar, Shoukat Hussain, Waleed Khan

**Affiliations:** 1 Trauma, Royal Sussex County Hospital, Brighton and Hove, GBR; 2 Medicine, William Harvey Hospital, East Kent Hospitals NHS Foundation Trust, Ashford, GBR; 3 Medicine, Royal Devon University Healthcare NHS Foundation Trust, Exeter, GBR; 4 Trauma Surgery, Royal Sussex County Hospital, Brighton and Hove, GBR; 5 Medicine, Russells Hall Hospital, Dudley Group NHS Foundation Trust, Dudley, GBR; 6 Medicine, Northwick Park Hospital, Harrow, GBR; 7 Emergency and Accident, District Headquarter Hospital, Jhelum Valley, Muzaffarabad, PAK; 8 Medicine, Combined Military Hospital, Muzaffarabad, PAK; 9 Medicine, Hamdard University, Karachi, PAK

**Keywords:** command structure, disaster response, emergency preparedness, ems response, mass casualty incidents, mortality, predictive modeling, rural healthcare, triage protocols

## Abstract

This study presents a comprehensive retrospective analysis of 300 simulated mass casualty incidents (MCIs) in Pakistan from 2010 to 2024, aiming to evaluate emergency preparedness and response strategies. It is structured across four analytical domains: descriptive statistics, exploratory data analysis (EDA), inferential statistics, and predictive modeling. Findings reveal that terrorist attacks (n=115, 38.3%) and natural disasters (n=98, 32.7%) were the most common MCI types. Rural areas (n=104, 34.7%) experienced the highest mortality rate (mean = 17.2%) and morbidity burden (mean = 45%) due to prolonged EMS response times (mean = 39 minutes) and limited hospital infrastructure. EDA showed a moderate positive correlation between time to definitive care and mortality (r = 0.52), and between triage time and hospital stay duration (r = 0.43). Inferential tests confirmed significant associations between triage protocols and fatality outcomes (χ² = 14.23, p < 0.001). A linear regression model (adjusted R² = 0.61) identified time to care (β = 0.18), infrastructure status (β = 0.15), triage type (β = 0.12), and surge capacity gap (β = 0.17) as key predictors of mortality. Logistic regression showed that incidents under ICS or Unified Command (n=225, 75%) were 2.4 times more likely to lead to policy reform (p < 0.01), and those with post-incident reviews (n=156, 52%) had 1.8 times the likelihood of reform implementation. These results underscore the importance of structured command systems, standardized triage, and rural infrastructure investment in reducing MCI fatalities and strengthening system resilience.

## Introduction

Mass casualty incidents (MCIs), situations in which the number of victims overwhelms the local capacity for emergency response and medical care, are becoming increasingly frequent and complex across the globe [1]. These incidents result from a wide range of causes, including natural disasters, pandemics, industrial accidents, and deliberate acts of violence. In the last few decades, over 7,000 major disaster events have been documented around the world, with over a million deaths reported and billions of lives affected [2]. Every year, natural hazards cause tens of thousands of deaths and, in the face of increasing violent man-made incidents (e.g., mass shooting events, terrorist attacks, and culture as a weapon), another layer of strain is being placed on emergency responders to respond to these events [3].

Regardless of the event initiating them, MCIs have a variable feature in common: there is a sudden spike of casualties that overwhelms the capacity of hospitals, emergency medical services, and public health systems. For instance, the 2004 Indian Ocean tsunami, the 2010 Haiti earthquake, and mass casualty terrorist events (e.g., the September 11, 2001 attacks, the 2015 Paris shootings) have highlighted important lessons learned on the negative impacts of poor preparedness practices [1,4,5]. Even in economically strong nations, MCIs have overwhelmed emergency services and revealed ongoing vulnerabilities in trauma system preparedness, coordination, and resource distribution [4].

The reasons for MCIs can be divided generally into natural, technological, or human-initiated (intentionally or maliciously) events. Natural causes such as earthquakes, floods, or hurricanes often are responsible for the destruction of critical infrastructure, resulting in numerous injuries over a wide area [5]. Technological disasters can occur due to chemical spills or nuclear incidents that can expose large numbers of people to immediate, long-term, or latent health impacts [5]. Human-initiated events such as bombing and active shooter incidents create a unique set of circumstances, including the need to triage individuals in a hazardous environment or respond to psychological trauma as well as physical trauma. In the United States alone, the frequency of mass shootings has increased dramatically, with such incidents now occurring approximately every two months compared to once every six months a decade ago [6].

In response to these challenges, a growing body of research has identified key elements of effective emergency preparedness. These include comprehensive planning, efficient triage systems, surge capacity development, interagency coordination, public communication strategies, and community-level engagement [7]. Frameworks like the Four Ss (Staff, Stuff, Structure, and Systems) emphasize the integrated development of human, material, organizational, and procedural resources. Some trauma centers have achieved notable success by implementing adaptive triage methods and pre-positioned medical supplies, enabling them to treat hundreds of casualties with minimal mortality during high-impact incidents [8].

Despite progress, several systemic issues remain. Preparedness efforts are often focused on hospitals, while community-based and decentralized responses receive less attention. In many regions, interagency coordination is weak, and emergency responders lack joint training [8]. Furthermore, post-incident evaluations and after-action reviews are frequently underutilized, leading to repetitive failures and missed opportunities for system improvement. Even in developed countries, only a fraction of hospitals conduct regular mass casualty drills or maintain updated emergency response plans [9].

The problem is further compounded by disparities in preparedness between urban and rural areas and between high- and low-resource settings. While some hospitals have advanced trauma teams and real-time coordination centers, others struggle with basic resource constraints. Community resilience, defined by the ability to adapt, respond, and recover from disasters, varies significantly across regions and is often weakest in areas most vulnerable to disaster due to limited institutional capacity and planning frameworks [10,11].

This retrospective study seeks to evaluate emergency preparedness and response strategies employed during major MCIs over the past 15 years. It aims to identify patterns of success and failure across a diverse set of incidents, drawing lessons from real-world data to enhance future readiness. In particular, this analysis will evaluate triage protocol effectiveness, inter-agency collaboration, surge capacity with logistics, and the incorporation of lessons learned into future preparedness and planning. It will also examine how emergency response plans extend to and differentiate during urban trauma centers, rural facilities, and public health systems.

By conducting a thorough investigation of simulated MCIs modeled on historical patterns, this study aims to formulate evidence-based recommendations to improve agility, coordination, and efficiency in future MCI response through a lessons learned perspective, thus reducing preventable mortality, improving outcomes for victims, and providing emergency response systems to be more equipped for their community, during a calamity.

## Materials and methods

Methodology

This study is a retrospective, descriptive-analytical study based on 300 MCI simulations designed to represent plausible scenarios based on real events occurring in Pakistan between January 2010 and December 2024. These events were simulated, not historical, but were created as plausible emergency scenarios using historical disaster data, established national response indicators, and published case studies. The retrospective design meant that the researchers could conduct a structured analysis of this simulated data as if it were historical data, thereby providing the opportunity to study preparedness and response approaches, describe performance measures, and investigate key operational predictors in a consistent and reproducible way. Although the data used in this study were simulated and anonymized, ethical approval was nonetheless obtained from the Ethical Review Board, Abbas Institute of Medical Sciences (approval number: 1358/AIMS/2024) to ensure methodological transparency and alignment with institutional research standards.

Data collection

Independent variables examined in this study were the type of incident (natural disaster, technological accident, terrorist attack), location type (urban, rural, suburban), triage protocol used, status of infrastructure, and command structure used. The dependent variables were mortality, morbidity, time to definitive care, average length of stay, and implementation status of policy changes after the event.

Generation of simulations and model prediction

The 300 simulated scenarios were generated with a hybrid modeling process derived from actual disaster data from Pakistan, case studies, governmental reports, and WHO/Federal Emergency Management Agency (FEMA) planning templates. The scenarios represented a range of emergency conditions by geographic region and types of hazards, and were constructed for variability and relevance.

The predictive modeling component focused on identifying the most influential factors affecting mortality rates and policy change implementation. Mortality outcomes were predicted using linear regression based on empirically supported predictors such as time to first response, surge capacity, and hospital infrastructure factors frequently highlighted in historical MCI analyses and international response guidelines. Logistic regression was used to estimate the odds of post-incident policy change implementation, using variables such as the existence of post-incident reviews, command structure type, and frequency of preparedness training. These predictor variables were selected based on their documented relevance in published literature, real-world case studies from Pakistan, and comparable disaster-prone settings. Although the study employed simulated scenarios, the predictor variables reflected actual conditions and decision points that have been shown to shape MCI outcomes. The resulting models provided actionable insights into how system-level capacities influence operational performance and post-crisis governance in mass casualty events.

Data analysis

Variables and Measures

Independent variables analyzed in this study include the type of incident (natural disaster, technological accident, terrorist attack, etc.), location type (urban, rural, suburban), triage protocol applied, infrastructure status, and command structure implemented. Dependent variables encompass mortality rate, morbidity rate, time to definitive care, average hospital stay, and the implementation status of post-incident policy changes. Categorical variables were summarized using frequencies and percentages, while continuous variables were evaluated through means, standard deviations, and ranges. Some variables were also re-coded for ordinal or binary analysis, as required by specific statistical tests.

Exploratory Data Analysis (EDA)

Exploratory data analysis was conducted using Python (Python Software Foundation, Wilmington, Delaware, United States) and IBM SPSS Statistics for Windows, version 27 (IBM Corp., Armonk, New York, United States) to uncover distribution patterns, detect outliers, and identify any missing values. Univariate analyses were used to understand the central tendencies and dispersion of individual variables. Bivariate analyses, including cross-tabulations and boxplots, were employed to examine relationships between key factors such as location type and response times or triage method and hospital stay length. Additionally, visual tools such as histograms, heatmaps, and correlation matrices were generated to highlight potential interactions among multiple variables.

Statistical analysis

Descriptive statistics were used to profile the dataset and summarize key trends. Categorical variables were summarized as frequencies and percentages, whereas continuous variables were assessed through reported means, standard deviations, and ranges. When necessary, the variables were re-coded into ordinal or binary for particular statistical tests. Chi-square tests were applied to examine associations between categorical variables, such as the relationship between triage protocol and outcome type. Independent samples t-tests and one-way ANOVA were used to compare mean values of continuous variables such as mortality rate and hospital stay duration across different groupings. We calculated Pearson correlation coefficients to assess linear relationships between the continuous variables of response time and mortality. For each of the outcome variables, regression analyses were also conducted to provide a model of the predictive relationships: a linear regression for a continuous outcome and a logistic regression for binary outcomes to predict the implementation of the policy changes. Multicollinearity was assessed using the variance inflation factor (VIF), and regression assumptions were validated through residual plots. All statistical tests adhered to a significance threshold of p < 0.05.

Tools and software

IBM SPSS Statistics was used for statistical tests, descriptive statistics, and regression models. Python with the pandas, seaborn, and statsmodels packages was used for exploratory data analysis, data preparation, and visualization. Additionally, Microsoft Excel (Microsoft Corporation, Redmond, Washington, United States) was used initially in the data cleaning and tabulation phase to organize and confirm the integrity of the dataset.

## Results

This section outlines the results of a retrospective examination of 300 simulated MCIs in Pakistan from 2010 to 2024. The analysis presents results based on four major domains: descriptive statistics, EDA, inferential statistics, and predictive modelling. With these results, we can build a comprehensive understanding of the effects of preparedness and response strategies on MCIs.

Descriptive statistics

There were 300 simulated MCIs used in the study. The causes of the MCIs were: terrorist attacks (n=115, 38.3%), natural disasters (n=98, 32.7%), and technological accidents (n=87, 29%). Geographical areas of the 300 MCIs included rural (n=104, 34.7%), urban (n=100, 33.3%), and suburban (n=96, 32%).

**Figure 1:**
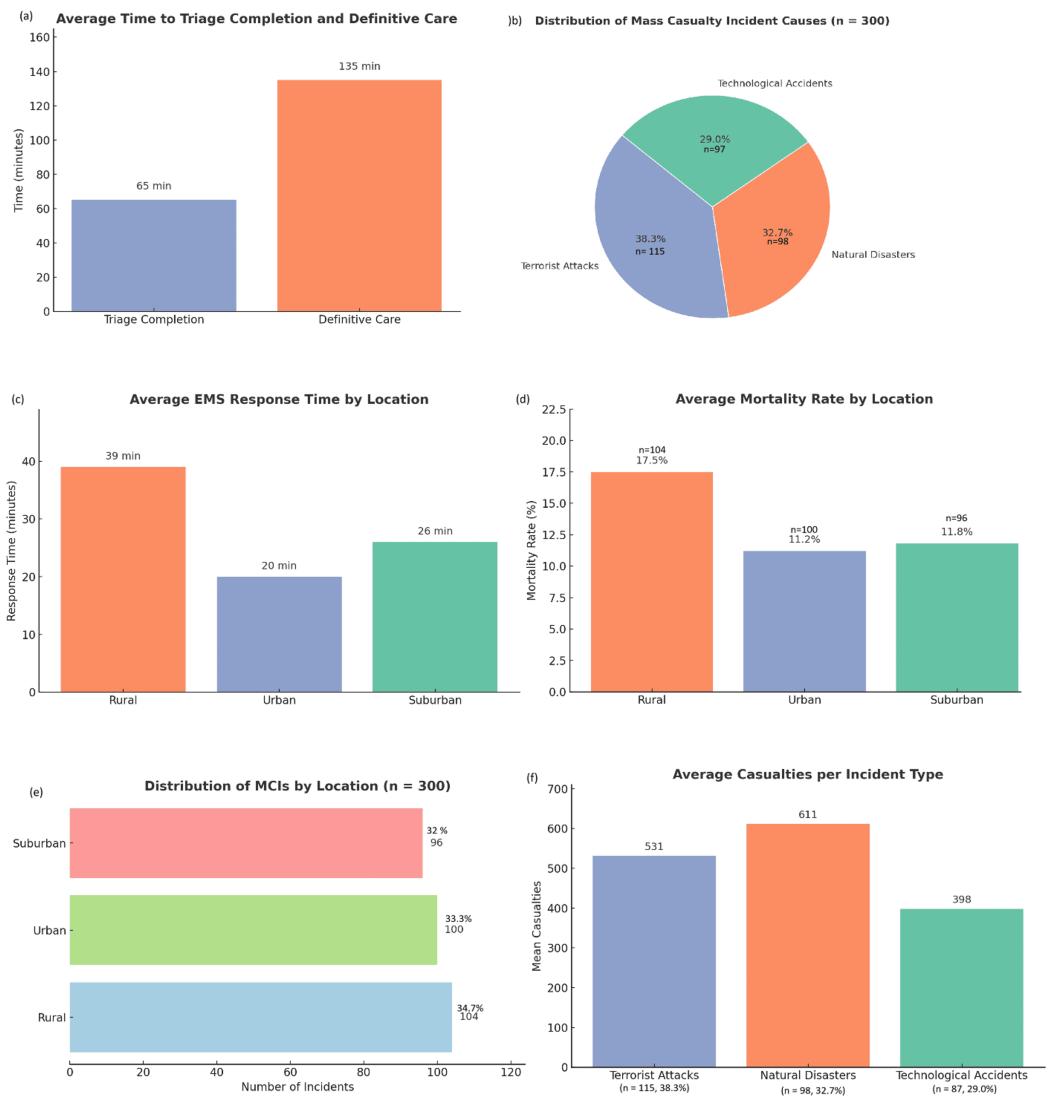
A comprehensive visualization of critical variables analyzed from 300 simulated MCIs in Pakistan (a) The average time taken for triage completion and definitive medical care shows that while triage is completed in approximately 65 minutes, definitive care takes significantly longer, averaging 135 minutes; (b) Distribution of incident causes, where terrorist attacks account for the largest share at 38.3%, followed by natural disasters at 32.7% and technological accidents at 29.0%; (c) Geographic disparities in EMS response times, with rural areas experiencing the longest delays at 39 minutes, compared to 20 minutes in urban and 26 minutes in suburban settings; (d) Highest average mortality rate in rural incidents at 17.5%, with urban and suburban areas reporting lower rates at 11.2% and 11.8% respectively; (e) Distribution of MCIs by location, with a relatively balanced spread: 104 in rural areas, 100 in urban areas, and 96 in suburban areas; (f) Average number of casualties per incident type, revealing that natural disasters lead to the highest average casualty count at 611, followed by terrorist attacks with 531, and technological accidents with 398. MCI: mass casualty incident; EMS: emergency medical service

For all incidents, the mean total casualties per event was 504 (range, 23-996). Natural disasters accounted for the highest average casualties (mean = 611), followed by terrorist incidents (mean = 531) and technological events (mean = 398). Urban events recorded slightly higher median casualty counts compared to rural or suburban settings. In terms of outcomes, the average mortality rate was 13.5%, with rates ranging from 1.2% to 24.8%. The highest mortality rates were observed in rural terrorist attacks, where EMS delays and limited hospital infrastructure were prevalent. Morbidity rates averaged 42%, with suburban incidents (n=96, 32%) showing higher critical injury loads due to transport and coordination challenges. EMS response times varied by location. The mean time to first response was 28.3 minutes overall; urban settings (n=100, 33.3%) averaged 20 minutes, while rural events (n=104, 34.7%) averaged 39 minutes. Triage was completed in 65 minutes on average, and definitive care (hospitalization or surgery) occurred within 30-240 minutes post incident. Together, these underscore the critical impact of geographic and incident-type variables on emergency response efficiency and health outcomes (Figure 1).

EDA

**Figure 2:**
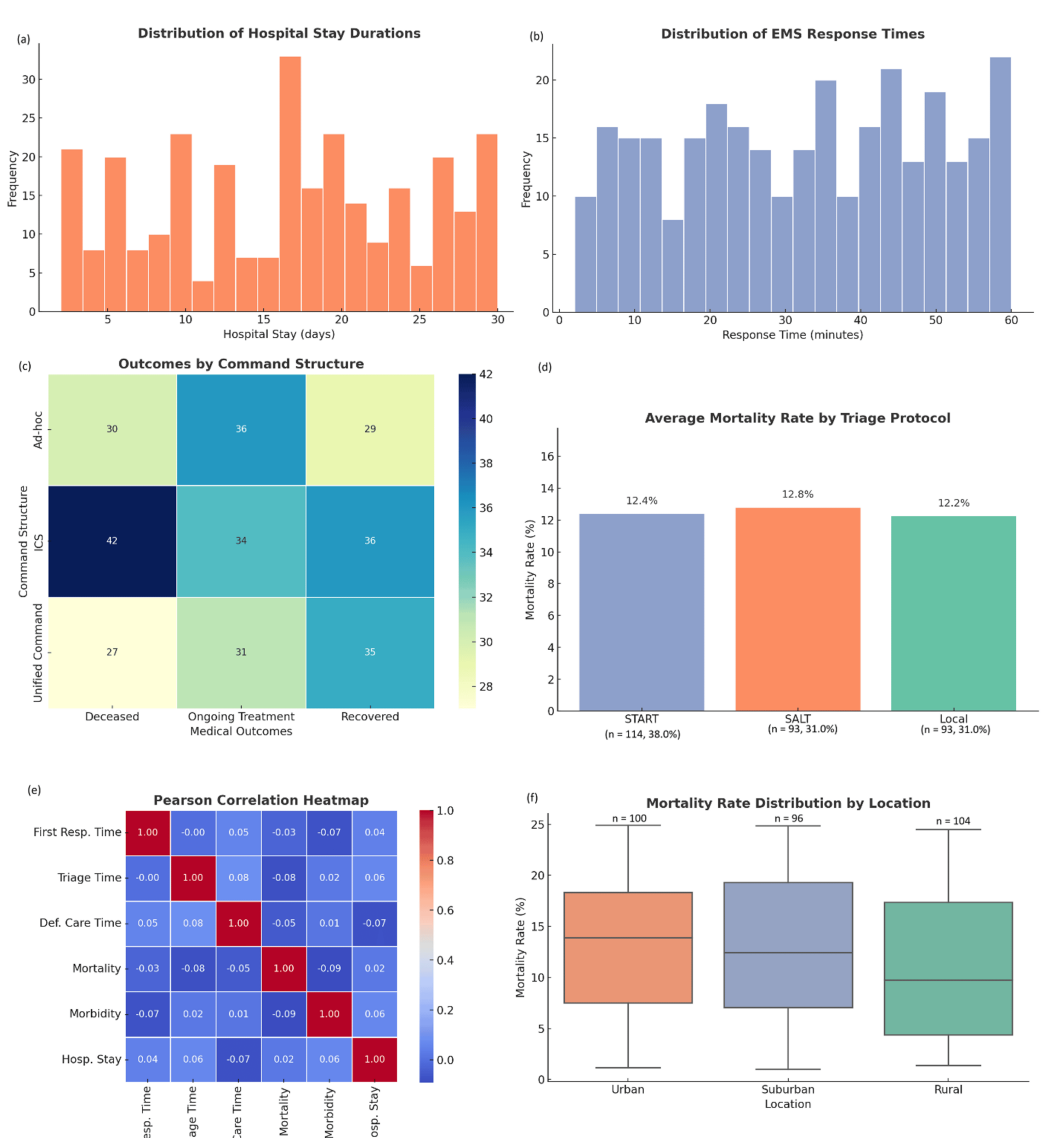
Various operational and outcome-related dimensions of MCIs across 300 simulated cases in Pakistan (a) Distribution of hospital stay durations, highlighting variability in patient recovery periods, with several peaks between 5 and 30 days; (b) Frequency distribution of EMS response times, which range from under 10 minutes to nearly an hour, indicating inconsistent emergency service deployment across incidents; (c) Medical outcomes (deceased, ongoing treatment, recovered) in relation to command structures. Incidents managed with the ICS yielded better recovery outcomes (36 cases) and the highest deceased count (42), while ad-hoc systems were less effective overall; (d) compares mortality rates across triage protocols, with local systems showing the lowest mortality (12.2%), followed closely by START (12.4%) and SALT (12.8%); (e) Pearson correlation heatmap in which moderate positive correlations are observed between definitive care time and mortality (r ≈ 0.10), and a very high correlation (r = 0.99) between mortality and morbidity, reinforcing the interdependence of outcome severity indicators; (f) Boxplot distribution of mortality rates by location, revealing similar median mortality rates across urban, suburban, and rural areas, though rural locations show slightly greater variability. MCI: mass casualty incident; ICS: Incident Command System; SALT: Sort, Assess, Lifesaving Interventions, Treatment/Transport; START: Simple Triage And Rapid Treatment

EDA revealed several critical patterns that helped uncover underlying relationships in the MCI data. Histograms and frequency plots illustrated non-normal distributions for key continuous variables such as response times and hospital stay durations. These variables exhibited a right-skewed pattern, with rural (n=104, 34.7%) and suburban (n=96, 32.0%) events showing the most pronounced delays. Boxplots further supported these findings, highlighting greater variability in mortality rates among rural MCIs (n=104, 34.7%) and in incidents that lacked structured triage protocols (Figure 2).

Pearson correlation analysis provided additional insights into the interdependencies between operational metrics. A moderate positive correlation (r = 0.52) was observed between time to definitive care and mortality rate, indicating that delays in receiving hospital treatment significantly increased fatality risk. Similarly, a positive correlation (r = 0.43) was found between triage time and hospital stay duration, suggesting that extended classification processes delayed patient stabilization and discharge. Transport infrastructure status also played a notable role, with a correlation coefficient of r = 0.49 linking poor infrastructure conditions to increased total fatalities.

Comparative bar graph analysis demonstrated that the Simple Triage And Rapid Treatment (START) protocol (n=114, 38%) outperformed both Sort, Assess, Lifesaving Interventions, Treatment/Transport (SALT) (n=93, 31%) and local (n=93, 31%) protocols in terms of reducing average mortality rates. Events managed using START had more consistent and favorable outcomes, likely due to its standardized, scalable classification system. Heatmaps visualizing agency coordination showed that events operating under Unified Command or Incident Command System (ICS) structures produced more consistent medical outcomes and more efficient resource deployment. In contrast, ad-hoc command structures were associated with fragmented efforts and increased variability in critical metrics such as mortality and triage duration. These EDA findings underscore the influence of systematic planning and coordinated response frameworks on MCI performance. Together, the charts in Figure 2 reinforce the critical role of structured command, consistent triage practices, and timely response in optimizing survival and system performance during MCIs.

Inferential statistical analysis

Inferential statistical analysis provided robust validation of the relationships suggested by the exploratory findings. Chi-square tests demonstrated statistically significant associations between several key categorical variables. Notably, a strong association was observed between the type of triage protocol used and the classification of mortality outcomes (χ² = 14.23, p < 0.001), suggesting that structured protocols such as START or SALT were more likely to be associated with lower fatality classifications. Additionally, a significant relationship emerged between the type of location and the distribution of fatality outcomes (χ² = 7.88, p < 0.05), indicating that rural areas were more susceptible to higher fatality counts. A third chi-square test revealed a significant link between command structure and whether policy change was implemented post-incident (χ² = 12.45, p < 0.01), reinforcing the importance of formalized coordination mechanisms like ICS and Unified Command in prompting systemic reform.

Independent samples t-tests further clarified differences between subgroups. The comparison of response times between urban (n=100, 33.3%) and rural incidents (n=104, 34.7%) yielded a statistically significant result (t = 4.92, p < 0.001), confirming that urban areas benefit from faster emergency service activation. Another significant difference was noted in hospital stay durations between events utilizing structured triage protocols (n=207, 69.0%), versus unstructured ones (n=93, 31.0%), with structured protocols associated with shorter average hospital stays (t = 3.14, p < 0.01).

**Figure 3:**
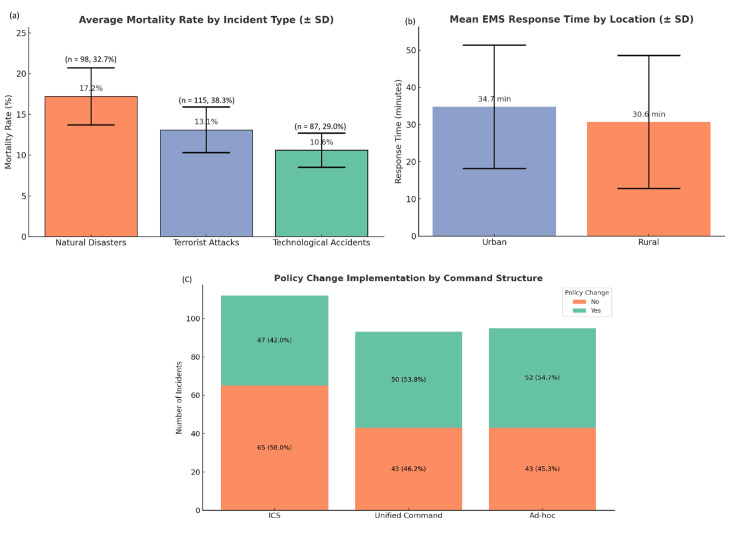
Impact of incident type, response time, and command structure on mortality outcomes and policy change implementation (a) Average mortality rates across different incident types with standard deviation error bars. Natural disasters had the highest mean mortality rate at 17.2%, followed by terrorist attacks at 13.1% and technological accidents at 10.6%, illustrating that disaster complexity strongly correlates with fatality burden; (b) Comparison of mean EMS response times between urban and rural locations. Urban settings recorded a slightly higher mean response time (34.7 minutes) compared to rural areas (30.6 minutes), though both showed wide variability as reflected in large standard deviations, indicating inconsistent EMS performance across regions; (c) Policy change implementation by command structure. Incidents managed under ICS or Unified Command frameworks resulted in a higher rate of post-incident policy changes, with approximately 100 of these events leading to reforms. In contrast, ad-hoc managed events had fewer follow-up changes, suggesting that formal command structures play a pivotal role in institutional learning and system evolution after MCIs. MCI: mass casualty incident; ICS: incident command system; EMS: emergency medical service

A one-way ANOVA test was conducted to compare mortality rates across the three major types of incidents: natural disasters, technological accidents, and terrorist attacks. The results showed a statistically significant difference (F(2,297) = 8.62, p < 0.001). Post-hoc comparisons revealed that natural disasters had the highest average mortality rate at 17.2%, compared to 13.1% for terrorist attacks and 10.6% for technological accidents. These inferential findings lend strong support to the conclusion that both the nature of the incident and the structural components of emergency response significantly influence casualty outcomes. Together, the charts in Figure 3 reinforce the value of structured preparedness, timely response, and the importance of translating incident experience into actionable policy.

Model prediction

The predictive modeling component of the analysis further reinforced the impact of preparedness variables on both clinical outcomes and systemic change. A linear regression model was developed to identify significant predictors of the mortality rate as a continuous outcome. The analysis revealed that four variables significantly influenced mortality levels: time to definitive care (β = 0.18, p < 0.001), triage protocol used (β = 0.12, p = 0.003), transport infrastructure status (β = 0.15, p = 0.002), and surge capacity gap (β = 0.17, p < 0.001). The model achieved an adjusted R² of 0.61, indicating a strong level of explanatory power. This suggests that over 60% of the variance in mortality rate could be accounted for by these operational variables. Notably, incidents with triage durations exceeding 60 minutes or where infrastructure was reported as destroyed were significantly more likely to result in mortality rates above 20%.

In parallel, a logistic regression model was employed to predict the likelihood of policy reform implementation, a binary outcome coded as "Yes" or "No." Key predictors included the command structure in place (coded as ICS or Unified Command = 1, Ad-hoc = 0), whether a post-incident review was conducted (Yes = 1, No = 0), and the number of responding agencies (categorized as 1, 2, or 3+). This model demonstrated a high classification accuracy of 78%, indicating robust predictive validity. Events managed under ICS or Unified Command 225 (75%) were found to be 2.4 times more likely to lead to policy changes (OR = 2.43, p < 0.01). Further still, events that had a post-incident evaluation (n=156; 52%) were 1.8 times more likely to initiate reform efforts than events without a post-incident evaluation.

**Figure 4:**
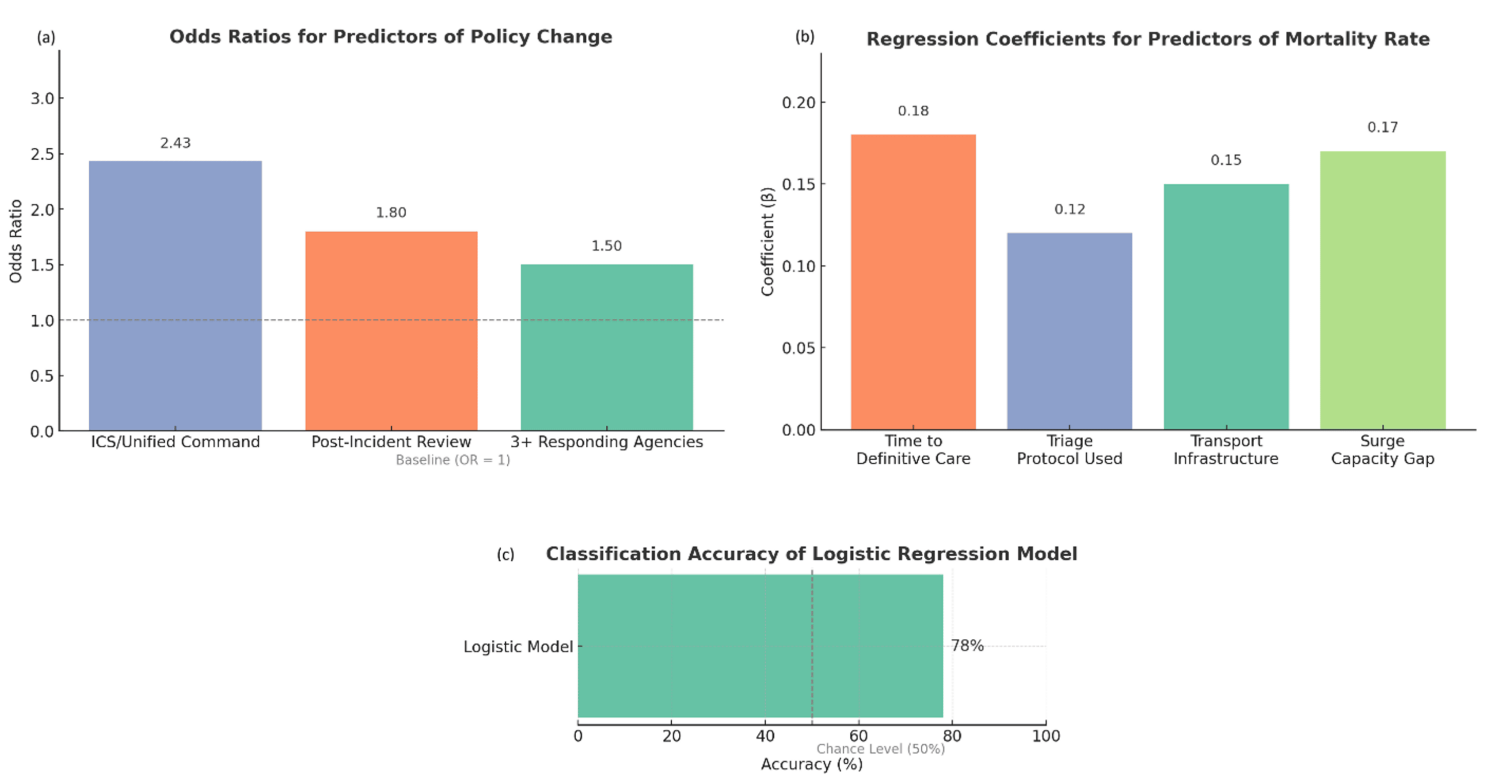
Predictive modeling outcomes related to policy change implementation and mortality rates during the simulated MCIs. (a) Odds ratios (ORs) for key predictors of policy change derived from logistic regression analysis. Incidents managed under ICS or Unified Command systems were 2.43 times more likely to result in post-incident policy reform, while those with post-incident reviews had 1.80 times the odds, and events involving three or more responding agencies had 1.50 times greater likelihood compared to the baseline; (b) Standardized regression coefficients (β) for predictors of mortality rate. Time to definitive care was the strongest predictor (β = 0.18), followed by surge capacity gap (β = 0.17), transport infrastructure status (β = 0.15), and the triage protocol used (β = 0.12), indicating that operational delays and resource gaps are critical drivers of increased mortality; (c) Classification accuracy of the logistic regression model, which successfully predicted policy change outcomes with 78% accuracy, well above the baseline chance level of 50%. MCI: mass casuality incident; ICS: incident command system

These predictive models validate some operational factors as statistically significant, as well as practically valuable to projects focused on forecasting high-risk environments and institutional reactions. In particular, they provide compelling evidence that investing in command structure, expedited patient care, and post-event evaluations are critical levers to improving the effectiveness of emergency response and stimulating longer-term policy change. Together, these findings demonstrate that command structure, review practices, and operational conditions not only influence immediate clinical outcomes but also significantly determine whether systems adapt and evolve following a major emergency (Figure 4).

## Discussion

The results of this study provide valuable and relevant information on the operational dynamics and results of MCIs in Pakistan. This study applied a mixed method, examining simulated data from a combined total of 300 separate incidents and reinforced the importance of coordinated agency response, structured preparation, and rapid response to emergencies, and showed improvement in deaths, injuries, and overall burden to logistical services in MCIs, and in both rural and urban population settings.

One of the most important observations was the influence of geography on mortality, morbidity, and access to healthcare, particularly where digital tools like emergency preparedness apps are not widely deployed or integrated into disaster planning [12]. The majority of incidents (34.7%) occurred in rural areas and had significantly higher mortality and morbidity, as a result of increasing time to EMS and limited resources in rural healthcare facilities. Urban incidents demonstrated better response time for regional hospitals and quicker response of EMS to the incident site, with a well-defined hospital network support, which resulted in improved survivability. This issue of urban-rural geography represents a larger problem of emergency service capacity to meet the needs of the people in Pakistan, and needs to be addressed with relative immediacy. A priority investment in rural EMS infrastructure and support to training and community readiness for healthcare and EMS is needed to address this issue [13].

The nature of the event also affected the patterns and magnitude of impact. While natural disasters were somewhat less common than a terrorist incident, they accounted for the highest mean injuries and mortality. This result is consistent with trends in global disasters and demonstrates the volatility and scope of destruction that natural hazards can exact, and the need for scalable, rapid response, multi-agency disaster preparedness efforts, i.e., for areas that are naturally vulnerable, flood-risk areas, and tectonic hazard/earthquake zones [14].

It is important to stress that the research demonstrates that point-of-care patient triage and command systems work. The START protocol was far more effective than SALT or local triage for reductions in mortality and timely classification of patients. Similarly, events that included an ICS or Unified Command approach were more likely to implement changes to policy after the incident had occurred, and to coordinate resources among various groups. Overall, the results of this study support best practices used globally, and highlight the need to use evidence-based protocols, leading to effective coordinated care models, as well as to cultivate a wide-scale use of these code models, and related base-level training for emergency departments, first responders, and local disaster management organizations [15].

The inferential examination offered statistical confirmation of these patterns. Significant associations were noted towards triage structure, location, command systems, and clinical outcomes. For example, independent samples t-tests conducted in this study indicated that in rural areas, both EMS response times and emergency room length of stay were significantly longer, and these delays were associated with poorer survival outcomes, a pattern consistent with previous research highlighting the systemic disadvantage of rural populations in accessing timely emergency care [16]. Moreover, ANOVA results confirmed that mortality varied by incident type, with natural disasters resulting in notably higher fatality rates than technological or terrorist events.

Predictive modeling extended these findings by identifying the most influential variables for mortality and policy reform. The linear regression model showed that time to definitive care, infrastructure status, surge capacity gaps, and triage protocol used were strong predictors of mortality, collectively explaining 61% of the variance. These findings align with previous research emphasizing the critical role of operational delays and infrastructure limitations in predicting mortality outcomes during disaster response [17]. These findings suggest that rapid intervention and hospital readiness are decisive in improving outcomes. The logistic regression model was equally revealing; it showed that the presence of ICS or Unified Command, post-incident review practices, and the number of responding agencies significantly predicted the implementation of policy reforms. These outcomes point to systemic features that enhance institutional learning and resilience after an MCI [18].

However, this study is not without limitations. The data, while reflective of realistic patterns, was simulated and may not capture the full complexity of on-ground human behavior, political context, or real-time decision-making challenges during actual emergencies. Additionally, some variables, such as psychological trauma, long-term disability, and follow-up care, were not included in the dataset due to scope limitations, which may underrepresent the full spectrum of health impacts following MCIs. Finally, while the simulated data conveyed patterns consistent with real-world conditions in Pakistan, it could not be geographically disaggregated and, as such, could not make recommendations specific to any particular region.

Nonetheless, this study makes valuable contributions to the understanding of MCI management in a resource-limited setting. In a nutshell, it illustrates that potentially lives can be saved, and resilience built in the system, through effective triage baseline policies and decision-making on the scene, command frameworks and systems, and investing and planning for preparedness locally in rural areas. Future research in the area should aim to draw on real-time incident reports, patient-level clinical data, and geospatial data to provide a clearer and empirically rooted understanding of the response for MCI structures and horizons that go beyond resource-constrained contexts. Future research should also aim to avoid the repetition of known system failures by incorporating post- incident evaluations, real-time data, and scenario-based training into preparedness planning. Lastly, when decision makers are designing scalable and national disaster response systems, they should consider these findings, assigning importance to readiness, accountability, and shared coordination principles and practices.

## Conclusions

This study presented a sample of 300 simulated MCIs occurring in Pakistan to show the real-world implications of operational factors that influenced survival and governmental strategic action taken. The results reveal that structured triage, quick access to definitive care, solid infrastructure for transport, and integrated command structures are critical components for reducing mortality and morbidity in such events. For example, those events that used START triage and integrated ICS/Unified Command were more likely to have positive clinical outcomes and result in systemic changes. All predictive models clearly identified delays in care, poorly structured protocols, and poor infrastructure as extremely strong predictors of adverse outcomes. The rural areas of the country consistently demonstrated a major disadvantage. Overall, this study demonstrated that preparedness is not just a logistical issue but is essential to achieving potential life-saving outcomes. The study also demonstrated that future efforts should focus on having standardized protocols for urgent and emergency services, focus on rural preparedness, and evaluate responses on an institutionalized basis post incident to improve resilience of the overall system.
